# Divergent variations in concentrations of chemical elements among shrub organs in a temperate desert

**DOI:** 10.1038/srep20124

**Published:** 2016-01-28

**Authors:** Mingzhu He, Xin Song, Fuping Tian, Ke Zhang, Zhishan Zhang, Ning Chen, Xinrong Li

**Affiliations:** 1 Shapotou Desert Research and Experiment Station, Cold and Arid Regions Environmental and Engineering Research Institute, Chinese Academy of Sciences, Lanzhou, 730000, China; 2 Key Laboratory of Stress Physiology and Ecology in Cold and Arid Regions of Gansu Province, Lanzhou, 730000, China; 3 Department of Environmental Sciences, Centre for Carbon, Water and Food, The University of Sydney, Camden, NSW 2570, Australia; 4 The Lanzhou Scientific Observation and Experiment Field Station of Ministry of Agriculture for Ecological System in the Loess Plateau Area, Lanzhou Institute of Husbandry and Pharmaceutical Sciences, Chinese Academy of Agricultural Sciences, Lanzhou, 730050, China

## Abstract

Desert shrubs, a dominant component of desert ecosystems, need to maintain sufficient levels of nutrients in their different organs to ensure operation of various physiological functions for the purpose of survival and reproduction. In the present study, we analyzed 10 elements in leaves, stems, and roots of 24 dominant shrub species from 52 sites across a temperate desert ecosystem in northwestern China. We found that concentrations of all 10 elements were higher in leaves than in stems and roots, that non-legumes had higher levels of leaf Na and Mg than did legumes, and that Na was more concentrated in C_4_ leaves than in C_3_ leaves. Scaling relationships of elements between the photosynthetic organ (leaf) and non-photosynthetic organs (stem and root) were allometric. Results of principal components analysis (PCA) highlighted the important role of the elements responsible for osmoregulation (K and Na) in water utilization of desert shrubs. Soil properties and taxonomy explained most variation of element concentrations in desert shrubs. Desert shrubs may not be particularly susceptible to future change in climate factors, because most elements (including N, P, K, Ca, Mn, Zn, and Cu) associated with photosynthesis, osmoregulation, enzyme activity, and water use efficiency primarily depend on soil conditions.

As a key component of desert ecosystems, desert shrubs not only play an essential role in the maintenance of ecosystem function and structure^1^,^2^, but also contribute significantly to nutrient cycling^3^, and account for much of the heterogeneous distribution of desert soil resources^4^,^5^,^6^. In order to survive in an environment of low water and nutrient availability, desert shrubs employ a variety of strategies to effectively improve uptake efficiency and/or reduce losses of water and nutrients; such strategies include but are not limited to deep rooting depths, low stomatal conductance, reduced levels of tissue nutrient concentrations, slow tissue turnover rates, and high nutrient resorption efficiency^7^. Notably, evolution has led to a diversification (but in a coordinated way) among different organs of desert shrubs in their functional roles in utilizing and/or acquiring nutrients in order to adapt to aridity and low nutrient availability. For example, for a photosynthetic organs such as leaf, sufficient levels of nutrient are required mainly for the purpose of sustaining relatively high levels of photosynthesis, high water use efficiency (WUE), and rapid growth during short periods of rain^2^,^8^; for non-photosynthetic organs such as stems and roots, their nutrient requirement would be different from that of leaves as they are designated to perform different functions, i.e., stems primarily function as a transportation and storage organ^9^ yet shrub roots are fundamental in water and nutrient uptake (as well as storage)^10^. However, although the intrinsic linkage of nutrient status to a specific physiological function has long been recognized, no study has comprehensively examined variations in nutrient levels among different organs of desert shrubs^11^,^12^, and the mechanism of element status in desert shrubs remains elusive.

Nitrogen (N) and phosphorus (P) constitute major elements of proteins and RNAs respectively^13^,^14^; yet both of these two essential nutrients are limiting in desert ecosystems^3^,^15^,^16^. Previous studies on nutrient allocation between metabolic and structural organs have largely focused on N and P stoichiometry i.e., 11,12,17. However, it is important to recognize that other elements also play essential roles in plant physiological functions^18^,^19^. For example, potassium (K) is an important activator for more than 60 enzymes, and regulates water relationships of osmosis, stomata opening, and transpiration^20^,^21^. Magnesium (Mg), a key component of chlorophyll, is involved in photosynthetic processes and the activation photosynthetic enzymes^13^. Calcium (Ca) maintains bio-membrane stability, which is critical for improving drought and heat resistance of desert plants^22^. Manganese (Mn), zinc (Zn), iron (Fe), and copper (Cu) play various roles in enzyme formation and act as catalysts in plant growth processes^13^. In desert alkaline soils, uptake of these elements is largely limited by soil pH and cation exchange capacity (CEC)^23^. Sodium (Na) is beneficial for halophytes because of its function in osmoregulation, but it is harmful for glycophytes due to its toxic ion effect^24^. Brownell *et al.* considered Na as a nutrient for some C_4_ species in the families Amaranthaceae, Chenopodiaceae, and Cyperaceae^25^, and Na may replace the function of K in saline environments^24^.

Plant nutrient levels are also known to vary according with N-fixation types (legume and non-legume) and photosynthetic pathways (C_3_ and C_4_ species)^8^,^13^. For example, compared to legumes, non-legumes generally exhibit lower N, but higher photosynthetic nitrogen-use efficiency (PNUE) and net photosynthetic rates (*A*)^26^. The higher PNUE and *A* reflect the fact that non-legumes tend to allocate a larger fraction of leaf N to carboxylation and bioenergetics, so as to enhances their ability to capture resources^27^. C_4_ species tend to have higher photosynthetic rates, WUE, and biomass accumulation than C_3_ species in dry and warm environments. However, in spite of the differences in their physiological performance, a recent survey study of flora in China found no significant differences in either N, P concentrations or N:P ratio between C_3_ and C_4_ herbs^28^. Additionally, our latest study indicated that C_4_ herbs of desert species concentrated more Mg, K, and N in shoots, which closely related to photosynthesis and osmoregulation, than C_3_ herbs of desert species^29^. It remains to be tested as to whether the conclusion drawn from herbaceous species can be extended to desert shrubs.

Previous studies have shown that factors potentially responsible for nutrient variations in plants include evolutionary history, environmental controls, and plant functional groups^11^,^12^,^17^,^18^,^30^,^31^. For example, Han, *et al.* demonstrated that plant functional groups is the most significant explanatory factor for the variation in leaf N, P, K, Ca, Mg, Fe, Mn, silicon (Si) and aluminium (Al), whereas climatic factors accounted for most of the variations in leaf sulphur (S) and Na^18^; Zhang, *et al.* showed that mean annual precipitation (MAP) and mean annual temperature (MAT) are more important than taxonomy in explaining leaf-level element variation^32^. Sardans *et al.* revealed that foliar N, P, K, Ca, and Mg of European forest tree species were co-determined by phylogenetic distances, climate, N deposition, forest types, and the nutrient niche of co-occurring species^33^. However, none of the above studies was focused on desert ecosystems. Previous authors have also examined scaling relationships of nutrients among different organs, but such examinations were largely restricted to a few elements such as N and P^12^.

The goal of the present study was to fill the knowledge gap concerning variations in element concentrations among different organs in desert plants. Toward this goal, we conducted an extensive field campaign in which 24 dominant shrub species were sampled from 52 sites across a temperate desert of northwestern China. For each sampled plant individual, we analysed 10 elements for mass-based concentration levels, for both photosynthetic (i.e., leaves) and non-photosynthetic (i.e., stems and roots) organs. With the collection of this comprehensive dataset, we aimed to test the following four hypotheses. Firstly, in this water and nutrient co-limited environment, we hypothesize that element concentrations of desert shrubs are higher in leaves than in stems and roots. Secondly, we hypothesize that variations in nutrient composition among desert shrubs can be a function of N-fixation types and photosynthetic pathways. Thirdly, we hypothesize that the scaling of element concentrations between the photosynthetic organ (leaf) and non-photosynthetic organs (stem and root) are allometric. Fourthly, in this regional study with relative narrow geographic scale, we hypothesize that soil and taxonomic factors explain most elemental variation among desert shrubs compared to climatic factors.

## Results

**Figure 1:**
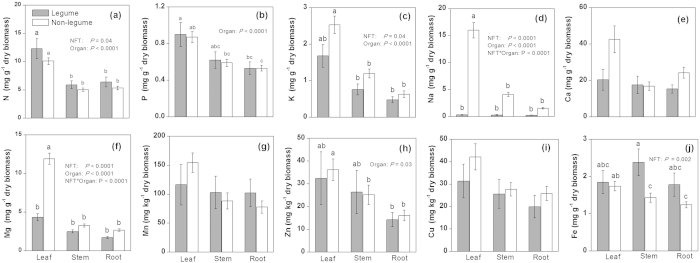
Mean ± standard error (error bars) of element concentrations in organs (leaf, stem and root ) of different N-fixation types (NFT, legumes and non-legumes). ANOVA *P*-values are reported when *P* < 0.05. Different letters above bars indicate significant differences of NFT and organs for each element (*P* < 0.05, Tukey’s HSD test).

**Figure 2:**
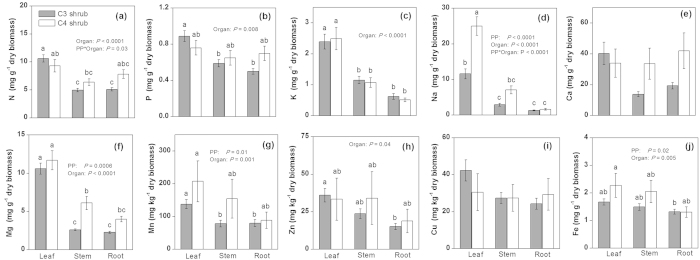
Mean ± standard error (error bars) of element concentrations in organs (leaf, stem and root ) of shrubs with different photosynthetic pathways (PP, C_3_ shrub and C_4_ shrub). ANOVA *P*-values are reported when *P* < 0.05. Different letters above bars indicate significant differences of PP and organs for each element (*P* < 0.05, Tukey’s HSD test).

Element concentrations of desert shrubs displayed considerable variations among plant organs (leaves, stems, and roots), N-fixation types, and photosynthetic pathways (Table 1, Figs 1 and 2). Concentrations of 10 elements analysed all exhibited significant variations among plant organs. Of the 10 elements, 8 (N, P, K, Na, Ca, Mg, Mn, and Cu) displayed higher concentrations in the photosynthetic organ (i.e., leaf ) than in the non-photosynthetic organs (i.e., stems and roots), whereas the rest 2 (Zn and Fe) had significantly higher concentrations in non-root organs (i.e., leaf and stem) than in roots (Table 1). With regard to N-fixation types, non-legume plants were found to have markedly higher concentrations in K, Na, Mg and Zn but lower levels in N and Fe when compared with legume species (Fig. 1). For Na and Mg, significant interactions were observed between N-fixation types and plant organs: there was no significant concentration differences between legumes and non-legumes in non-photosynthetic organs (stems or roots); but this is not true for leaves, for which concentrations of both elements were significantly higher in non-legumes than in legumes (Fig. 1). Further, we found significant differences in concentrations of Na, Mg, Mn and Fe between plants having different photosynthetic pathways (Fig. 2). Interactions of between photosynthetic pathway and plant organ were significant for N and Na; in particular for Na, leaf concentration was ca. 2.5-fold higher in C_4_ than in C_3_ shrubs yet root and stem concentrations did not differ significantly between the two photosynthetic types (Fig. 2).

The scaling relationships of element concentrations across different organs revealed some variations that were dependent on specific organ pairs involved (Table 2). For all 10 elements, slopes for the reduced major axis (RMA) regressions of leaves vs. stems and leaves vs. roots (except Fe between leaves and stems) were all significantly larger than 1, this is an indication for allometric scaling where element concentrations increased faster in leaves than in stems and roots. The slope for stem vs. root relationships were significantly larger than 1 for the case of P, K, Na, Mg, Mn, Zn, and Fe; for the rest of the elements (Ca, Cu and N) isometric scaling (i.e. slope was not statistically different from 1) were found between stems and roots (Table 2).

**Figure 3:**
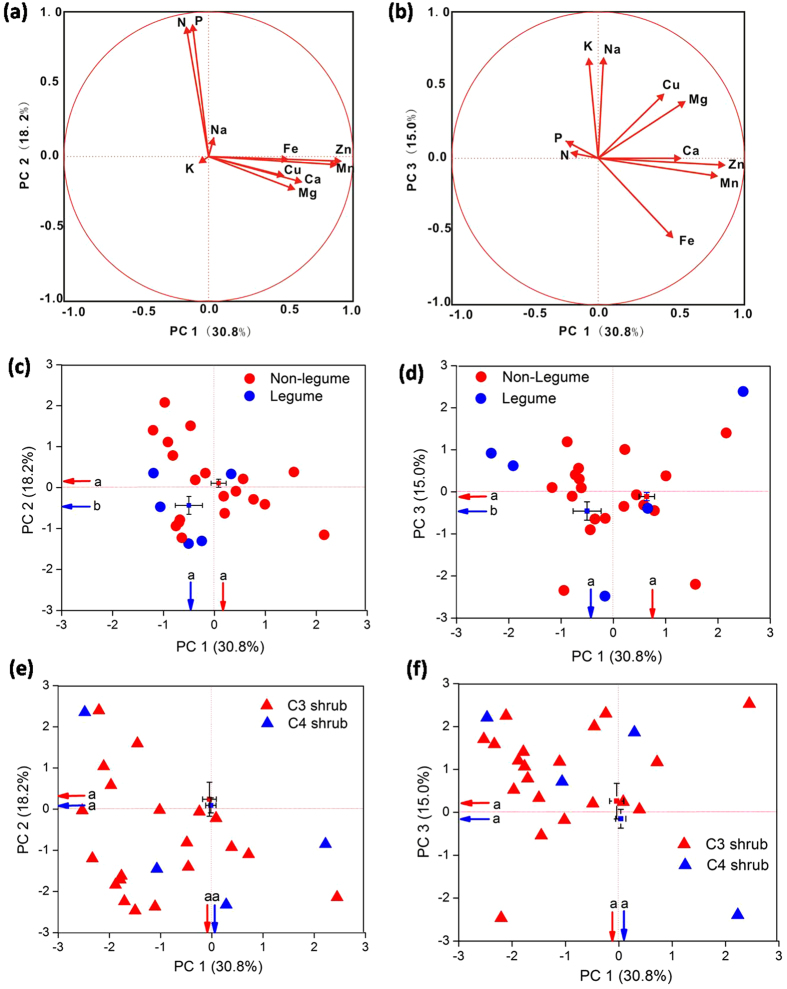
Principal component (PC) analysis showing (a) loading values of 10 leaf elements for PC axis 1 and 2 and (**b**) PC axis 1 and 3; and (**c**) score plots between legumes and non-legumes (species level) along PC axis 1 and 2 and (**d**) PC axis 1 and 3; and (**e**) score plot between C_3_ and C_4_ shrubs (species level) along PC axis 1 and 2 and (**f**) PC axis 1 and 3. Arrows (in blue and red) indicate the values of the mean of coordinate scores of different N-fixation types (legume and non-legume shrubs) and photosynthetic pathways (C_3_ and C_4_ shrubs) in the PC axis 1, 2 and 3. Different letters indicate significant differences (*P* < 0.05). Error bars show standard error (legume, n = 29; non-legume, n = 169; C_3_ shrub, n = 164; C_4_ shrub, n = 30).

Using PCA analysis, the factor loading of ten elements was different among leaves, stems, and roots (Table 3). Ca, Mg, Mn, Zn, Cu, and Fe in leaves, stems, and roots loaded mainly on the first PC axis, which explained 30.8%, 40.9%, and 34.7% of the total variability, respectively (Table 3, Fig. 3a,b). Leaf N and P, stem N and P, and root K and Na loaded mainly on the second PC axis, which explained 18.2%, 18.7%, and 21.4% of the total variability, respectively (Table 3, Fig. 3a). The third axis was loaded by leaf K and Na, stem K and Na, and root N and P, which explained 15.0%, 15.1%, and 17.6% of the total variability, respectively (Table 3, Fig. 3b). Across the PC axis 2 and 3, the scores of non-legume shrubs were significantly higher than those of legume shrubs (Fig. 3c,d). There were no significant differences between C_3_ and C_4_ shrubs across the PC axis 1, 2 and 3 (Fig. 3e,f).

Partial general linear models (GLM) were performed to test the effects of taxonomy, climate, and soil properties on elements concentrations. We found that full models explained a high portion of the variances in element concentrations of shrub leaves (Table 4), stems, and roots (Supporting information, Table S4). For leaf elements, the full model accounted for 46.1 to 85.6% of the total variability, whereas taxonomy and soil factors alone explained 2.89 to 37.3% and 9.9 to 48.2% of the total variations, respectively; climate only explained 0.002 to 2.76% of the variations. In addition, the rankings for the explanatory powers were not always consistent among the 10 elements; for example, with regard to leaf Na, Mg, and Fe, taxonomy was the most important factor in explaining the concentration variations, yet for leaf N, P, K, Ca, Mn, Zn, and Cu, soil properties were the most critical explanatory factors (Table 4). For stem and root elements, the models exhibited similar explanatory power as for leaves and explained 41.3 to 79.4% and 27.0 to 74.2% of the total variances, respectively (Table S4). More of the variations were also explained by taxonomy and soil properties than by climate variables. Independent effects of taxonomy explained most of the total variations in stem Na and Fe, and root Na, Mg and Fe, while soil properties accounted for most of the total variations in stem N, P, K, Ca, Mg, Mn, Zn, Cu, and root N, P, K, Ca, Mn, Zn, Cu (Table S4).

## Discussion

We found support for our first hypothesis that desert shrubs tend to have higher element concentrations in leaves than in stems and roots (Table 1). There are two possible explanations for such a pattern. Firstly, leaves perform a number of physiological functions (such as photosynthesis, respiration, and water utilization) that are vital to plant survival and productivity in particular under extreme conditions such as desert environments; as such they would require high levels of nutrients to ensure normal operation of these functions^1^,^8^. Secondly, desert shrubs generally invest a large fraction of biomass to belowground organs for the purpose of acquisition of water and nutrients; the high fraction of biomass in roots (and stems) inherently indicates that a substantial amount of nutrients were stored in these organs, but when evaluated by concentration, large biomass is a diluting factor that can lead to lower concentrations of elements in these organs (i.e. when compared with leaves). Notably, our results are consistent with Yang *et al.* who also found that shrub species in arid regions of Northern China had higher nutrient concentrations in leaves than in non-photosynthetic organs^12^.

In the present study, we found that elemental concentrations were higher in leaves than in non-leaf organs; nevertheless, when compared with their counterparts reported for average Chinese flora, leaves of the desert shrubs in our study were lower in concentrations of several elements^18^,^32^; these elements include N, P, K, Mn, Zn, and Cu. By contrast, levels of Ca, Mg, Na and Fe in leaves of desert shrubs were higher than those of Chinese flora. This latter pattern is interesting given that the availability of most elements in desert soil is low (i.e., as a result of low solubility and low absorption efficiency).We speculate that higher concentrations of Mg, Na, Ca, and Fe in shrub leaves may be tightly linked to the need for high WUE, photosynthesis, and other basic physiological functions of desert plants^14^, but more work needs to be done to corroborate this. Generally, Mg is a component of chlorophyll, and plays an important role in photosynthesis and enzyme activation^13^. Mg is preferentially bound to N and P groups, and accumulation of a larger pool of Mg may provide support for optimum photosynthesis of desert shrubs when water is available.

We also found support for our second hypothesis that variations in nutrient concentration among desert shrubs can be a function of N-fixation types and photosynthetic pathways. With respect to N-fixation types, only N, K, Na, and Mg concentrations were significantly different among leaves, stems, and roots and between legume and non-legume shrubs (Fig. 1a,c,d,f). Na concentrations among leaves, stems and roots in non-legumes were significantly higher than in legumes. Previous studies indicated that many legumes are sensitive to a high accumulation of Na and exclusion of excess Na from the developing organs keeps the cytoplasm from experiencing Na toxicity^14^. In this study, most of the non-legumes were halophytes due to high levels of Na concentrations in the soil and high pH (Supporting information, Table S3), and previous studies indicated that growth of many halophytes can be enhanced when there are high Na concentrations in the substrate (generally, 10–100 mM Na, but up to 510 mM Na in extreme cases^34^). In desert saline soil with low availability of K^3^, Na is generally more accessible and can substitute for some of the functions of K; for example, accumulation of Na in non-legume organs can significantly improve their osmoregulation and WUE. Mg concentrations in leaves of non-legumes were higher than those in legumes (Fig. 1f), this is consistent with the fact that non-legumes tend to have higher photosynthetic capacity than legumes^35^. Fe has low solubility in alkaline soils and is difficult for desert plants to use. In the present study, we found that Fe concentrations in legumes were significantly higher than that in non-legumes (Fig. 1). This might be due to that legume roots can generally cause soil to be more acid, an effect resulting from net H^+^ efflux from the roots during the N_2_ fixation process^14^,^36^, thereby promoting the absorption of Fe by legume roots. We also found that leaf Na of C_4_ shrubs was significantly higher than that of C_3_ shrubs (Fig. 2d). Generally, shrubs with C_4_ pathways display higher photosynthetic rate, WUE, and biomass production, especially under arid and hot environmental conditions^34^,^37^,^38^. Previous studies have demonstrated that without adequate Na, some C_4_ species grew poorly and exhibited visual deficiency symptoms including chlorosis and necrosis, or failure to form flowers^25^,^39^. Resupplying Na^+^(100 uM) alleviated these visual symptoms and improved growth. Therefore, Brownell *et al.* considered Na an important nutrient for C_4_ species in the families Chenopodiaceae, Cyperaceae, and Amaranthaceae^25^. To date, we have no clear idea how Na affects metabolism and fine structure in the mesophyll chloroplasts of C_4_ species^13^. At the very least, greater accumulation of Na in leaves can improve WUE of C_4_ shrubs under water-limited conditions.

Our third hypothesis, that the scaling relationships of element concentrations between the photosynthetic organ (leaf) and non-photosynthetic organs (stem and root) are allometric, was also supported. According to the RMA regression results, we found regression slopes (RSs) of elements in leaves vs. stems (except Fe), leaves vs. roots, and stems vs. roots (except N, Ca and Cu) were larger than 1, and element concentrations in the upper organs increased faster than in the lower organs (Table 2). The observed allometric relationships between leaves and the non-leaf organs is a further indication that more nutrients are needed for shrub leaves to conduct basic physiological activities (such as photosynthesis, biomass accumulation, and production of flowers and seeds) during the short rainy seasons in desert environments. Interestingly, we also found that RSs of Na and Mg in leaves vs. stems (3.37 and 3.34) and in leaves vs. roots (10.5 and 4.66) were larger than for other elements (Table 2). This provided evidence for the vital roles Na and Mg play in photosynthesis and WUE for desert shrubs, as we discussed earlier. Na is key element for osmoregulation and perhaps plays an more important role than K in this temperate desert. To date, most previous studies focused on the scaling relationships of N and P among different organs. Based on the published seed plant data, Kerkhoff, *et al.* calculated the RSs of N and P in stems vs. leaves (1.38 and 1.39) and roots vs. leaves (1.33 and 1.38) of woody plants, which indicated that more N and P were allocated to stems and roots than to leaves^11^. Yang, *et al.* also found allometric relationships for N and P concentrations for roots vs. leaves and stems vs. leaves in shrub flora across Northern China^12^. These results are consistent with Brouwer’s hypothesis that plant organs closer to the source of nutrients will be more successful than distant organs^40^. However, Brouwer’s hypothesis was not supported by our result, as we found higher N and P levels in leaves than in stems and roots. The unique pattern of ours alternatively suggested that nutrient utilization strategies for shrubs in desert ecosystems are such that photosynthetic organs (leaves) must maintain higher nutrient concentrations than non-photosynthetic organs (stems and roots) in order to guarantee sufficient photosynthetic activity and water utilization after rainfall events.

Element convergence in different organs of desert plants contributes to their biochemical function and physiological properties^41^. For leaf elements (Table 3, Fig. 3), we found that the first PC axis represented the major variances for most metal elements (including Ca, Mg, Mn, Zn, Cu, and Fe), which represented the “structural and enzymatic” element set^42^. Ca is a component for cell wall stabilization, Mg is found in chlorophyll and benefits ribosome structure, Mn helps maintain the structure of lamellar membrane systems of chloroplasts, and Zn, Cu, and Fe are actors or components of enzymes^9^,^13^. The second PC axis represented the “nucleic acid—protein” element set, which included N and P. N and P are associated with the metabolism of proteins, nucleic acids, and amino acids. The third PC axis represented the “osmoregulation” element set and is associated positively with Na and K, both of which are important for osmotic adjustment and WUE. As such, our PCA results are mostly consistent with those of the previous studies^32^,^42^,^43^; one exception is that an “osmoregulation” element set was identified in our study, highlighting the importance of water utilization for desert shrubs.

**Figure 4:**
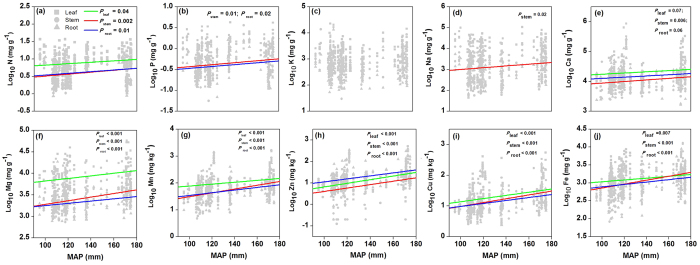
Relationships of mean annual precipitation (MAP) with element concentrations (N, P, K, Na, Ca, Mg, Mn, Zn, Cu and Fe) among leaves, stems and roots. Colored lines represent significant relationships (*P* < 0.5) for shrub organ (green, leaves; red, stems; blue, roots).

Our last hypothesis, that soil and taxonomic factors are more important than climatic factors in explaining the variations of element concentrations across desert shrubs, was also supported. According to the partial GLM results, we found that the percentage of elemental variations explained by three factors (taxonomy, climate, and soil) combined varied for different organs: 46.1 to 85.6% of the variation was explained for leaves (Table 4), 41.3 to 79.4% for stem (Supporting information, Table S4), and 27.0 to 74.2% for roots (Table S4). Soil and taxonomy factors had greater explanatory power than climate, which for example only explained 0.002 to 2.76% of the variation in leaf elemental concentrations (Table 4). In this regional study, MAP and MAT under arid conditions had relatively small variations, with ranges of 97–175 mm and 6.2–9.3 °C, respectively. Presumably as a result of the magnitude of variation, MAT was not a significant explanatory factor for most of the elements (Na and Mg were the only exception; Supporting information, Fig. S1). However, we found that correlation with MAP was significant for 7 elements, including N, Ca, Mg, Mn, Zn, Cu, and Fe (Fig. 4a,e–j). It can be argued that the observed significant influence of MAP on shrub element concentrations is a reflection of the general importance of soil moisture on nutrient availability of desert soils. Indeed, our further analysis indicated that element concentrations of shrub organs mostly depended on soil water conditions, for example, soil water content (SWC) at 20–40 cm depth had significant effects on all leaf elements, while leaf N, P, and Mg were significantly affected by SWC at 0–10 cm depth and leaf N, P, Mg, Zn, and Cu were significantly affected by SWC at 40–100 cm depth (Supporting information, Table S5).

In conclusion, nutrient levels and physiological characteristics in desert shrubs determine their survival abilities and functioning. The result of our study showed that desert shrubs have greater element concentrations in leaves than in stems and roots, and that nutrient accumulation rates in leaves were faster than in stems and roots (allometric relationship). We observed higher concentrations of Na and Mg in non-legumes than in legumes, and higher concentration of Na in C_4_ shrubs than in C_3_ shrubs, thus indicating that Na may play an equal or more important role than K in osmoregulation of desert shrubs. Spatial variations in element concentrations across our sampling sites were mainly accounted for by taxonomy and soil properties, with climatic factors only playing a minor role. As such, we conclude that desert shrubs may not be particularly susceptible to future change in climate factors as most elements (including N, P, K, Ca, Mn, Zn, and Cu) associated with photosynthesis, osmoregulation, enzyme formation and WUE primarily depend on soil conditions.

## Materials and Methods

### Site description

There were a total of 52 vegetated sites chosen for sampling in this study. All sampling sites were dominated by desert shrubs, and situated within the Alxa Desert which is a temperate desert located in northwestern China (Supporting information, Table S1) and covering a range in latitude from 37°39′N to 40°39′N and longitude from 101°11′E to 105°43′E. Across the sampling sites, mean annual temperature (MAT) varied between 6.2 and 9.3 °C and mean annual precipitation (MAP) between 95 to 175 mm. In this region, desert soils are sandy, saline (with high Na^+^concentrations and electrolytic conductivity), alkaline (pH range of 7.5 to 11.2), and exhibit low nutrient availability^44^.

### Field survey and sampling

Field surveys and plant and soil sample collections were conducted during the growing season of 2012 (August). Based on the vegetation map of Inner Mongolia^44^, we initially identified the community distribution of dominant desert shrubs. At the time of our field surveys and sample collection, all sampling sites were free of grazing activities and anthropogenic disturbances. Quantitative survey of the vegetation was carried out at each site to record species composition, life form, richness, and height. Meanwhile, we recorded geographical coordinate and elevation of each site using GPS (eXplorist 500, Magellan, USA). We randomly collected soil samples using a hand auger (made by a polyvinyl chloride tube) from three soil layers (0–20 cm, 20–40 cm, and 40–100 cm) with three replicates. Soil samples were mixed evenly and stored in plastic bags and subsamples for measurements of soil moisture were put into aluminium cups with sealed caps. Soil water content (SWC, w/w %) was measured by gravimetric method on the same day. For plant samples, 5 individuals of each shrub were selected and dug up with roots to a soil depth of 100 cm. Plant samples were divided into leaves, stems, and roots, and at least 5 replicates of the different organs were combined. In total, we collected 582 plant samples belonging to 8 families and 24 shrub species, which included 5 legume and 19 non-legume species, and 4 C_4_ and 20 C_3_ species (Supporting information, Table S2), and 156 soil samples.

### Chemical analysis

Leaf, stem, and root samples were rinsed with deionised water to remove dust and soil, oven-dried at 60 °C for 72 h, and then finely milled before measurement of element concentrations. Nitrogen concentration in leaves, stems, and roots were measured using a CHNS/O Elemental Analyzer (Perkin Elmer, USA). Phosphorus in plant samples was determined colorimetrically after H_2_SO_4_-H_2_O_2_-HF digestion with the ammonium molybdate/stannous chloride method^45^.

Soil samples were homogenized and air-dired. After manual removal of stones, roots and other debris, soil samples were finely milled for measurement of elements. Soil total nitrogen (SN) was measured by a Kjeltec system 2300 Analyzer Unit (Tecator, Höganäs, Sweden). Soil total phosphorus (SP) content was determined using the molybdate/ascorbic acid blue method after digestion with HClO_4_ and H_2_SO_4_ acid^46^. The soil pH was measured by a pH meter (PHSJ-3F, China) using a water extraction method (10 g fresh soil extracted with 50 ml water). Electrolytic conductivity (EC) was determined by a portable conductivity meter (Cole-Parmer Instrument Company, USA).

The total concentration of K, Na, Ca, Mg, Mn, Zn, Cu, and Fe were measured through different digestion procedures for plant samples (ultrapure concentrated HNO_3_ 8 ml) and soil samples (ultrapure concentrated mixture of HNO_3_ (2.5 ml) + HF (4 ml) + HCl (1.5 ml) ). All samples were placed in 50 ml Teflon centrifuge tubes and then solubilised and digested in a microwave oven (Multiwave 3000, Anton Paar GmnH, Austria). Meanwhile, we measured blank solutions (acid mixture without sample) in duplicate during each group of sample digestions. Standard samples, polar leaves (GBW 07604) and agricultural soil (GBW E070045) (China Standard Reference Materials Centre), were used to assess the precision and accuracy of the digestions and analytical procedures. After digestion, the concentrations of metal elements in plant and soil samples were measured by inductively coupled plasma (ICP-OES 7000DV, Perkin Elmer, USA). In this study, MAT and MAP were considered climatic variables and were obtained using linear interpolation models based on latitude, longitude, and altitude, which were derived from the climate database from the Inner Mongolia Weather Bureau (Table S1).

### Data analysis

We calculated the descriptive statistics (mean, standard error, coefficient of variation) of element concentrations in leaf, stem, and root of 24 desert shrubs, N-fixation type (legume and non-legume), and photosynthetic pathway (C_3_ and C_4_ species). We used a one-way ANOVA to test effects of plant organ on element concentrations across all sites. When effects of plant organ were significant (*P* < 0.05), we used Tukey’s HSD posthoc test to compare means of the plant organs. We also used two-way ANOVA to test effects of both N-fixation type and plant organ, both photosynthetic pathway and plant organ, and their interactions on element concentrations.

To examine scaling relationships of elements among leaves, stems, and roots, we used reduced major axis regression (RMA) performed by the SMART package (http://www.bio.mq.edu.au/ecology/SMART). First, we used a scaling approach, *Y* = a*X*^b^ (*Y*, concentration of element in leaf or stem; *X*, concentration of element in stem or root) to examine allometric relationships. After log-transformation, the power function became a linear regression equation, where *a* and *b* were the regression intercept and slope, respectively^12^. Second, we considered the scaling relationship between *Y* and *X* as isometric when the 95% confidence interval (CI) of *b* included 1. Otherwise, the relationship was allometric, namely, that *Y* increased faster than *X* when *b* was above 1, whereas *Y* increased slower than *X* when *b* was below 1.

For soil properties, we conducted a one-way ANOVA to examine differences among the three soil layers. Because no significant differences (except for SWC and Ca) were found (Table S3), we recalculated the soil properties (except for SWC and Ca) as the weighed means of the three soil layers. Therefore, for each sampling site, we have three SWC and Ca values and 11 values for other soil properties (pH, EC, N, P, K, Na, Mg, Mn, Zn, Cu, and Fe). Principal component analysis (PCA) was conducted to examine whether element concentration in leaves, stems, and roots could be discriminated at the species level. We also performed a partial general linear model (GLM) (for details, see Heikkinen *et al.*^47^ and Han *et al.*^18^) to explore the independent and interactive effects of different factors on the variance of element concentrations in shrub organs. The total variance for each element was separated into taxonomy (family), climatic (MAT and MAP), and edaphic (soil properties) factors. All data were log-transformed to normalize the distribution of element concentrations among leaves, stems, and roots. All analyses were performed using JMP (v. 10.0.0; SAS Institute, Cary, NC, USA) and R 3.2.1(R Development Core Team, 2015).

## Additional Information

**How to cite this article**: He, M. *et al.* Divergent variations in concentrations of chemical elements among shrub organs in a temperate desert. *Sci. Rep.* **6**, 20124; doi: 10.1038/srep20124 (2016).

## Supplementary Material

Supplementary Information

## Figures and Tables

**Table 1:** Concentrations of analyzed elements in organs of desert shrubs.

Organ		Elements									
	Statistic	N (mg g^−1^)	P(mg g^−1^)	Ca(mg g^−1^)	Mg(mg g^−1^)	K(mg g^−1^)	Na (mg g^−1^)	Mn(mg kg^−1^)	Zn(mg kg^−1^)	Cu(mg kg^−1^)	Fe(mg kg^−1^)
Leaf	Mean	10.4a	0.87a	39.3a	10.7a	2.40a	13.6a	148.7a	35.6a	40.5a	1757.3a
n = 194	SE	0.58	0.05	6.35	0.63	0.21	1.29	15.2	4.35	5.08	116.1
	CV	77.1	82.9	225.2	82.2	124.2	131.5	141.2	170.2	174.8	92.1
Stem	Mean	5.51b	0.59b	22.8b	3.14b	1.14b	3.52b	90.3b	25.3ab	27.4b	1573.4ab
n = 194	SE	0.28	0.04	2.12	3.14	0.11	0.38	12.4	3.87	2.73	116.5
	CV	74.6	88.6	174.7	84.2	130.9	151.0	191.7	212.7	139.1	103.1
Root	Mean	5.18b	0.53b	16.9c	2.53c	0.60c	1.35b	81.3b	15.8b	25.0b	1318.5b
n = 194	SE	0.28	0.03	2.59	0.14	0.08	0.12	9.59	2.07	2.85	80.2
	CV	71.9	79.1	158.2	75.1	179.6	126.6	164.3	182.9	158.6	84.7
ANOVA result	F	53.0	19.5	7.82	137.5	40.4	70.9	8.41	7.71	5.03	4.35
	*P*	**<0.0001**	**<0.0001**	**0.0004**	**<0.0001**	**<0.0001**	**<0.0001**	**0.0002**	**0.0005**	**0.007**	**0.01**

Different letters indicate significant statistical differences among organs (Turkey’s HSD test, ANOVA, *P* < 0.05). *P*-values are in bold when *P* < 0.05. SE, standard error; CV, coefficient of variation; n, sample size.

**Table 2:** Summary of reduced major axis (RMA) regression results among leaves, stems, roots for each element.

	Nutrient	RS	95%CI	R^2^	*P*	n
Leaves vs. stems	N	**2.08**	1.90–2.28	0.58	<0.0001	194
	P	**1.37**	1.26–1.49	0.67	<0.0001	194
	K	**2.00**	1.77–2.27	0.26	<0.0001	194
	Na	**3.37**	3.02–3.75	0.42	<0.0001	194
	Ca	**3.00**	2.64–3.40	0.19	<0.0001	194
	Mg	**3.34**	2.94–3.79	0.19	<0.0001	194
	Mn	**1.22**	1.14–1.30	0.78	<0.0001	194
	Zn	**1.13**	1.06–1.20	0.80	<0.0001	194
	Cu	**1.86**	1.70–2.04	0.59	<0.0001	194
	Fe	1.00	0.90–1.10	0.53	<0.0001	194
Leaves vs. roots	N	**2.05**	1.87–2.25	0.57	<0.0001	194
	P	**1.72**	1.55–1.90	0.51	<0.0001	194
	K	**2.74**	2.46–3.06	0.39	<0.0001	194
	Na	**10.48**	9.28–11.9	0.26	<0.0001	194
	Ca	**2.45**	2.19–2.74	0.36	<0.0001	194
	Mg	**4.66**	4.08–5.31	0.14	<0.0001	194
	Mn	**1.58**	1.46–1.72	0.67	<0.0001	194
	Zn	**2.10**	1.94–2.27	0.69	<0.0001	194
	Cu	**1.78**	1.60–1.99	0.40	<0.0001	194
	Fe	**1.45**	1.27–1.65	0.18	<0.0001	194
Stems vs. roots	N	0.97	0.89–1.06	0.62	<0.0001	194
	P	**1.26**	1.15–1.37	0.61	<0.0001	194
	K	**1.37**	1.20–1.56	0.11	<0.0001	194
	Na	**3.11**	2.78–3.50	0.33	<0.0001	194
	Ca	**0.81**	0.77–0.87	0.83	<0.0001	194
	Mg	**1.40**	1.25–1.56	0.38	<0.0001	194
	Mn	**1.30**	1.18–1.43	0.55	<0.0001	194
	Zn	**1.86**	1.73–2.02	0.70	<0.0001	194
	Cu	0.96	0.90–1.02	0.79	<0.0001	194
	Fe	**1.45**	1.30–1.63	0.35	<0.0001	194

Regression slope (RS) estimates in bold are significantly different from 1, indicating the allometric relationships of leaf versus root for the related nutrient. CI, confidence interval; n, sample size.

**Table 3:** The factor loading of elements in leaf, stem, root of desert shrubs on the principal components analysis (PCA) axes at species level (N = 194).

	Leaf			Stem			Root		
	PC1	PC 2	PC 3	PC 1	PC 2	PC3	PC 1	PC 2	PC3
N	−0.163	**0.910**	0.081	−0.051	**0.930**	0.065	−0.122	0.009	**0.893**
P	−0.117	**0.928**	0.021	−0.029	**0.920**	0.015	−0.039	−0.003	**0.909**
K	−0.033	−0.010	**0.705**	−0.065	−0.018	**0.733**	0.019	**0.900**	−0.029
Na	0.029	0.127	**0.676**	0.099	0.076	**0.855**	−0.103	**0.890**	0.018
Ca	**0.649**	−0.184	−0.008	**0.909**	−0.112	0.078	**0.897**	0.065	−0.094
Mg	**0.601**	−0.233	0.340	**0.794**	0.034	0.387	**0.602**	0.671	−0.011
Mn	**0.892**	−0.046	−0.122	**0.968**	−0.034	−0.058	**0.859**	−0.014	−0.100
Zn	**0.926**	−0.032	−0.040	**0.932**	−0.099	−0.058	**0.926**	0.087	−0.136
Cu	**0.529**	−0.138	0.419	**0.518**	−0.314	0.137	**0.526**	0.236	−0.301
Fe	**0.569**	−0.029	−0.483	**0.737**	0.175	−0.231	**0.633**	−0.147	0.099
Total variation explained	30.8%	18.2%	15.0%	40.9%	18.7%	15.1%	34.7%	21.4%	17.6%

**Table 4:** Summary of the (partial) general linear models for the effects of taxonomy, climate, and soil factors on leaf element concentrations.

Element	Total effects (*r*^2^, %)				Independent and interactive effects (*r*^2^,%)						
	Full	Climate	Taxonomy	Soil	a.	b.	c.	ab	ac	bc	abc
N	64.3	25.2	36.3	52.2	0.15	2.89	**27.3**	6.10	0.54	8.89	18.5
P	76.6	43.4	50.7	44.8	0.002	5.05	**25.8**	26.8	0.04	2.27	16.6
K	57.7	17.1	35.5	37.2	0.75	13.2	**19.8**	6.54	1.67	7.61	8.15
Na	57.7	10.6	37.2	24.4	0.07	**25.2**	20.2	8.02	0.20	1.71	2.32
Ca	46.1	4.84	15.4	34.5	0.39	9.20	**29.9**	2.05	0.38	2.17	2.02
Mg	85.6	18.1	55.1	41.7	2.40	**37.3**	26.0	4.26	2.18	4.22	9.29
Mn	68.0	18.7	27.1	51.9	2.04	6.78	**37.0**	7.25	1.91	5.55	7.49
Zn	76.8	16.1	24.7	61.1	2.76	7.16	**48.2**	5.86	1.20	5.43	6.24
Cu	53.7	12.0	25.2	37.0	0.06	10.2	**28.4**	6.55	0.07	3.21	5.31
Fe	47.5	15.6	34.1	22.7	1.97	**17.6**	9.90	5.20	1.48	4.39	6.91

In the partial GLM, leaf element variations were partitioned into different components: (i) a, b, c denote the independent effects of climate, taxonomy, and soil, respectively; (ii) ab, ac, and bc are respectively the shared effects between climate and taxonomy, climate and soil, and taxonomy and soil, minus abc; (iii) abc represent the shared effects of climate, taxonomy and soil together (for details, refer Heikkinen *et al.*^46^ and Han *et al.*^18^). Climatic variables: MAP and MAT; soil factors: pH, EC, SWC in 0–20 cm, 20–40 cm, and 40–100 cm, weighted averages of soil N, P, Mg, K, Na, Mn, Zn, Cu, and Fe.
