# Supplementary material for: Divergent variations in concentrations of chemical elements among shrub organs in a temperate desert
Source: Sci Rep. 2016 Jan 28;6:20124. doi: 10.1038/srep20124 (PMC4730183; doi:10.1038/srep20124)
Supplement: Supplementary Information [file srep20124-s1.pdf]

Subject areas: Ecosystem ecology; Biogeochemistry

Title:

Divergent variations in concentrations of chemical elements among shrub organs in a temperate desert

Mingzhu He[1,2], Xin Song[3], Fuping Tian[4], Ke Zhang[1,2], Zhishan Zhang[1,2], Ning Chen[1,2], Xinrong Li[1,2]

[1]Shapotou Desert Research and Experiment Station, Cold and Arid Regions Environmental and Engineering Research Institute, Chinese Academy of Sciences, Lanzhou, 730000, China

[2]Key Laboratory of Stress Physiology and Ecology in Cold and Arid Regions of Gansu Province, Lanzhou, 730000, China

[3]Department of Environmental Sciences, Centre for Carbon, Water and Food, The University of Sydney, Camden, NSW 2570, Australia

[4]The Lanzhou Scientific Observation and Experiment Field Station of Ministry of Agriculture for Ecological System in the Loess Plateau Area, Lanzhou Institute of Husbandry and Pharmaceutical Sciences, Chinese Academy of Agricultural Sciences, Lanzhou, 730050, China

Author for correspondence:

Mingzhu He

Tel: +86 931 4967193

Email: hmzecology@lzb.ac.cn

Table S1 Study sites in Alxa desert

Plot	Altitude(m)	Longitude	Latitude	MAP (mm)	MAT(^oC)	Shrub Species
No.1	37°39.00′	105°00.00′	1279	170.8	9.3	*Nitraria tangutorum*, *Kalidium cuspidatum*, *Slenderbranch Kalidium*
No.2	37°41.42′	104°59.52′	1302	170.5	9.1	*Sarcozygium xanthoxylon, Nitraria tangutorum*
No.3	37°43.23′	104°58.42′	1338	171.3	9.0	*Sarcozygium xanthoxylon, Reaumuria soongorica, Oxytropis aciphylla, Ammopiptanthus mongolicus*
No.4	37°46.43′	104°55.30′	1412	171.2	8.5	*Sarcozygium xanthoxylon, Nitraria tangutorum, Amygdalus mongolica*
No.5	37°52.02′	104°59.47′	1437	171.1	8.3	*Artemisia ordosica*
No.6	37°52.32′	105°07.03′	1503	174.6	8.0	*Sarcozygium xanthoxylon, Nitraria sphaerocarpa*
No.7	37°57.06′	105°21.11′	1326	171.1	8.7	*Nitraria tangutorum, Reaumuria soongorica, Slenderbranch Kalidium*
No.8	38°25.41′	105°43.28′	1514	172.7	7.3	*Asparagus gobicus, Reaumuria soongorica, Salsola passerina*
No.9	40°11.46′	104°48.46′	1316	121.7	7.3	*Sarcozygium xanthoxylon, Potaninia mongolica, Ammopiptanthus mongolicus, Ceratoides lateens, Salsola passerina*
No.10	40°16.64′	104°45.69′	1313	119.4	7.3	*Sarcozygium xanthoxylon, Reaumuria soongorica,Oxytropis aciphylla, Potaninia mongolica, Salsola laricifolia,Ceratoides latens, Salsola passerina*
No.11	40°19.88′	104°45.08′	1280	117.3	7.4	*Nitraria tangutorum*
No.12	40°34.73′	104°34.61′	1294	110.8	7.2	*Caragana brachypoda, Sarcozygium xanthoxylon, Nitraria tangutorum, Reaumuria soongorica, Potaninia mongolica,Ceratoides lateens, Salsola passerina*
No.13	40°39.77′	104°33.77′	1257	108.0	7.4	*Sarcozygium xanthoxylon, Reaumuria soongorica, Oxytropis aciphylla, Potaninia mongolica, Nitraria sphaerocarpa, Ceratoides latens*
No.14	40°14.46′	104°33.25′	1366	118.9	7.1	*Nitraria tangutorum, Reaumuria soongorica, Potaninia mongolica, Ceratoides latens, Salsola passerina*
No.15	40°13.65′	104°15.22′	1462	117.9	6.7	*Sarcozygium xanthoxylon, Nitraria tangutorum, Reaumuria soongorica, Potaninia mongolica, Ceratoides lateens, Salsola passerina*
No.16	40°09.91′	104°01.38′	1421	114.8	7.1	*Sarcozygium xanthoxylon, Nitraria tangutorum, Oxytropis aciphylla, Caragana Korshinskii, Ceratoides latens*

No.17	39°53.04′	103°38.46′	1511	117.6	6.9	*Caragana brachypoda, ,Sarcozygium xanthoxylon, Asparagus gobicus, Reaumuria soongorica, Oxytropis aciphylla, Brachanthemum mongolicum, Potaninia mongolica, Ceratoides lateens, Convolvulus gortschakovii*
No.18	39°28.76′	102°54.91′	1275	108.6	8.6	*Reaumuria soongorica, Potaninia mongolica, Nitraria sphaerocarpa, Convolvulus gortschakovii, Salsola passerina*
No.19	39°24.58′	102°42.32′	1261	106.7	8.8	Nitraria sphaerocarpa, Convolvulus gortschakovii
No.20	39°24.88′	102°28.64′	1348	106.1	8.4	*Sarcozygium xanthoxylon, Nitraria sphaerocarpa, Artemisia desterorum*
No.21	39°23.27′	102°15.02′	1540	109.1	7.5	*Caragana brachypoda, Sarcozygium xanthoxylon, Oxytropis aciphylla, Brachanthemum mongolicum, Artemisia desterorum, Ceratoides latens*
No.22	39°19.90′	101°58.01′	1556	106.8	7.5	*Sarcozygium xanthoxylon, Oxytropis aciphylla, Artemisia desterorum*
No.23	39°18.98′	101°56.29′	1580	107.4	7.4	*Reaumuria soongorica, Brachanthemum mongolicum, Nitraria sphaerocarpa, Salsola passerina, Sarcozygium xanthoxylon*
No.24	39°12.01′	101°33.91′	1413	99.8	8.5	*Slenderbranch Kalidium*
No.25	39°12.66′	101°27.60′	1420	98.7	8.4	*Nitraria tangutorum*
No.26	39°12.66′	101°27.60′	1420	98.7	8.4	*Nitraria tangutorum, Slenderbranch Kalidium*
No.27	39°18.95′	101°11.87′	1463	95.1	8.3	*Nitraria sphaerocarpa, Slenderbranch Kalidium, Salsola passerina*
No.28	38°49.97′	101°33.84′	1846	117.0	6.8	*Reaumuria soongorica, Nitraria sphaerocarpa, Salsola passerina*
No.29	38°51.49′	101°33.72′	1778	116.7	6.9	*Reaumuria soongorica, Potaninia mongolica, Nitraria sphaerocarpa Salsola passerina*
No.30	38°57.04′	101°36.56′	1562	109.4	7.9	*Nitraria tangutorum, Reaumuria soongorica, Slenderbranch Kalidium*
No.31	39°02.64′	101°57.02′	1422	108.1	8.4	*Caragana brachypoda, Convolvulus ammannii, Anabasis brevifolia, Reaumuria soongorica, Potaninia mongolica, Nitraria sphaerocarpa, Salsola passerina*
No.32	38°56.68′	102°16.35′	1344	112.0	8.8	*Nitraria tangutorum, Sarcozygium xanthoxylon, Ammopiptanthus mongolicus, Caragana brachypoda, Ceratoides latens*
No.33	38°49.24′	102°22.59′	1339	115.6	8.9	*Potaninia mongolica, Reaumuria soongorica, Salsola passerina*
No.34	38°39.81′	102°16.86′	1439	120.1	8.5	*Salsola passerina, Reaumuria soongorica, Potaninia mongolica*
No.35	39°06.74′	102°33.48′	1536	118.2	7.6	*Salsola passerina, Potaninia mongolica, Sarcozygium xanthoxylon, Convolvulus gortschakovii*

No.36	40°06.87′	104°53.44′	1354	125.4	7.2	*Reaumuria soongorica, Potaninia mongolica, Sarcozygium xanthoxylon, Nitraria sphaerocarpa, Oxytropis aciphylla, Salsola passerina*
No.37	39°55.73′	104°58.32′	1564	136.0	6.2	Reaumuria soongorica, Oxytropis aciphylla, Convolvulus gortschakovii, Nitraria sphaerocarpa, Sarcozygium xanthoxylon
No.38	39°48.63′	105°03.97′	1477	137.0	6.7	*Sarcozygium xanthoxylon, Ammopiptanthus mongolicus, Asparagus gobicus, Caragana brachypoda*
No.39	39°36.98′	105°07.54′	1286	135.9	7.8	*Sarcozygium xanthoxylon, Ceratoides latens, Oxytropis aciphylla, Reaumuria soongorica*
No.40	39°33.35′	105°24.02′	1095	135.1	8.7	*Reaumuria soongorica, Salsola passerina*
No.41	39°24.98′	105°40.72′	1129	142.4	8.6	*Asparagus gobicus, Reaumuria soongorica*
No.42	39°14.59′	105°40.71′	1243	148.9	8.1	*Salsola passerina, Brachanthemum mongolicum*
No.43	39°06.64′	105°39.59′	1340	154.0	7.7	*Reaumuria soongorica, Kalidium cuspidatum, Salsola passerina*
No.44	38°41.35′	105°38.53′	1430	164.2	7.6	*Reaumuria soongorica, Salsola passerina*
No.45	38°41.19′	105°40.50′	1514	167.2	7.2	*Salsola passerina,Reaumuria soongorica, Asparagus gobicus*
No.46	38°41.33′	105°41.58′	1567	168.9	6.9	*Caragana tibeca, Caragana brachypoda, Oxytropis aciphylla, Ceratoides latens*
No.47	38°40.97′	105°42.49′	1633	171.1	6.6	*Reaumuria soongorica, Caragana brachypoda, Oxytropis aciphylla, Caragana tibeca*
No.48	38°40.95′	105°43.19′	1667	172.3	6.4	*Reaumuria soongorica*
No.49	38°23.81′	105°43.46′	1539	173.9	7.2	*Sympegma regelii, Salsola passerina*
No.50	38°23.31′	105°43.16′	1559	174.6	7.1	*Sympegma regelii*
No.51	38°15.27′	105°37.98′	1505	174.4	7.5	*Potaninia mongolica, Nitraria sphaerocarpa, Salsola passerina, Ceratoides latens*
No.52	38°06.52′	105°30.07′	1489	174.9	7.8	*Asparagus gobicus, Anabasis brevifolia, Salsola passerina, Potaninia mongolica, Sympegma regelii, Slenderbranch Kalidium, Reaumuria soongorica*

Table S2 Species list of desert shrubs in Alxa desert

Species	Family	C3/C4 species	Legume/Non-legume
Ammopiptanthus mongolicus	Fabaceae	C3	Legume
Amygdalus mongolica	Rosaceae	C3	Non-legume
Anabasis brevifolia	Chenopodiaceae	C4	Non-legume
Artemisia desterorum	Asteraceae	C3	Non-legume
Artemisia ordosica	Asteraceae	C3	Non-legume
Asparagus gobicus	Liliaceae	C3	Non-legume
Brachanthemum mongolicum	Asteraceae	C3	Non-legume
Caragana brachypoda	Fabaceae	C3	Legume
Caragana Korshinskii	Fabaceae	C3	Legume
Caragana tibeca	Fabaceae	C3	Legume
Ceratoides latens	Chenopodiaceae	C3	Non-legume
Convolvulus ammannii	Convolvulaceae	C3	Non-legume
Convolvulus gortschakovii	Convolvulaceae	C3	Non-legume
Kalidium cuspidatum	Chenopodiaceae	C3	Non-legume
Nitraria sphaerocarpa	Zygophyllaceae	C3	Non-legume
Nitraria tangutorum	Zygophyllaceae	C3	Non-legume
Oxytropis aciphylla	Fabaceae	C3	Legume
Potaninia mongolica	Rosaceae	C3	Non-legume
Reaumuria soongorica	Tamaricaeae	C3	Non-legume
Salsola laricifolia	Chenopodiaceae	C4	Non-legume
Salsola passerina	Chenopodiaceae	C4	Non-legume
Sarcozygium xanthoxylon	Zygophyllaceae	C3	Non-legume
Slenderbranch Kalidium	Chenopodiaceae	C3	Non-legume
Sympegma regelii	Chenopodiaceae	C4	Non-legume

Table S3 Soil property in three soil depths

Soil property	Soil depth				
	0-20 cm	20-40 cm	40-1000 cm	F	*P*
pH	10.0±0.12	10.0±0.14	10.1±0.15	0.14	0.87
EC (ms.cm^{-1})	1.89±1.41	2.18±1.56	2.18±1.56	0.01	0.99
SWC (w/w%)	**2.63±0.30b**	**4.82±0.57a**	**4.75±0.58a**	6.19	**0.003**
SN (mg g-1)	0.21±0.02	0.19±0.02	0.19±0.02	0.27	0.76
SP (mg g-1)	0.66±0.04	0.60±0.04	0.58±0.04	0.97	0.38
Ca (mg g-1)	**5.90±0.36b**	**7.23±0.50ab**	**7.66±0.61a**	3.38	**0.04**
Mg (mg g-1)	3.85±0.20	3.96±0.21	3.96±0.23	0.08	0.93
K (mg g-1)	6.31±0.62	6.32±0.61	6.30±0.62	0.0003	1.00
Na (mg g-1)	7.39±0.50	7.29±0.46	7.40±0.49	0.01	0.99
Mn (mg kg-1)	439.6±22.6	410.6±20.2	419.0±22.5	0.47	0.63
Zn (mg kg-1)	33.2±2.44	30.1±2.21	30.5±2.57	0.51	0.60
Cu (mg kg-1)	14.9±0.79	14.8±0.81	14.6±0.95	0.05	0.95
Fe (mg g-1)	17.0±0.74	16.4±0.80	16.1±0.93	0.34	0.71

Different letters (a, b) indicate significant statistical differences between organs (Turkey's HSD test, ANOVA, $P < 0.05$). EC, electronic conductivity; SN, soil total nitrogen; SP, soil total phosphorus. Three soil depths are 0-20cm, 20-40cm and 40-100cm respectively.

Table S4 Summary of the (partial) general linear models for the effects of taxonomy, climate, and soil factors on leaf element concentrations

Organ	Element	Total effects (r^2, %)				Independent and interactive effects (r^2,%)						
		Full	Climate	Taxonomy	Soil	a.	b.	c.	ab	ac	bc	abc
Stem	N	79.4	37.5	59.4	47.6	0.21	9.77	**19.6**	21.8	0.19	12.5	15.4
	P	78.4	39.4	49.5	47.1	0.11	6.57	**28.7**	24.6	0.01	3.67	14.7
	K	41.3	6.71	13.5	33.5	0.43	5.83	**26.5**	1.53	0.84	2.23	3.91
	Na	49.7	10.2	34.9	24.3	0.01	**19.8**	14.6	5.59	0.15	5.06	4.44
	Ca	59.9	13.6	22.7	43.8	0.84	8.38	**35.6**	6.91	0.83	2.35	5.02
	Mg	56.4	14.2	35.4	31.6	1.13	18.3	**18.8**	5.32	1.09	5.06	6.68
	Mn	63.4	18.3	36.2	40.8	0.91	11.9	**25.5**	9.78	0.88	7.71	6.72
	Zn	73.3	23.0	36.5	52.7	2.80	8.98	**33.1**	8.85	0.99	8.29	10.3
	Cu	70.3	8.83	13.6	63.2	0.05	2.51	**56.4**	4.47	0.24	2.56	4.07
	Fe	55.5	13.0	47.6	19.0	0.29	**30.7**	7.39	5.46	0.22	4.32	7.04
Root	N	74.2	31.5	47.4	54.5	1.69	8.63	**23.7**	9.40	1.47	10.4	18.9
	P	68.3	24.7	32.8	50.4	0.16	4.96	**34.9**	12.8	0.44	3.76	11.3
	K	27.0	15.6	18.1	15.7	0.36	1.63	**8.53**	9.32	0.04	1.26	5.9
	Na	45.3	17.6	33.7	19.4	0.15	**14.3**	11.0	11.5	0.43	2.40	5.57
	Ca	55.2	13.4	21.6	39.6	1.09	7.77	**31.5**	6.74	1.08	2.58	4.50
	Mg	42.1	8.71	21.0	24.6	0.89	**12.0**	19.4	4.64	0.78	1.98	2.40
	Mn	54.4	15.1	23.5	38.9	0.98	7.00	**29.0**	7.51	0.94	3.34	5.65
	Zn	72.4	17.5	20.7	58.7	3.23	3.87	**47.0**	6.63	1.47	4.07	6.15
	Cu	65.5	10.6	14.2	59.8	0.02	3.06	**51.2**	2.66	0.15	0.72	7.74
	Fe	47.5	15.6	34.1	22.7	1.97	**17.6**	9.90	5.20	1.48	4.39	6.91

In the partial GLM, leaf element variations were partitioned into different components: (i) a, b, c denote the independent effects of climate, taxonomy, and soil, respectively; (ii) ab, ac, and bc are respectively the shared effects between climate and taxonomy, climate and soil, and taxonomy and soil, minus abc; (iii) abc represent the shared effects of climate, taxonomy and soil together. Climatic variables: MAP and MAT; soil factors: pH, EC, SWC in 0-20 cm, 20-40 cm, and 40-100cm, weighted averages of soil N, P, Mg, K, Na, Mn, Zn, Cu, and Fe.

Table S5 Statistic results of the general linear models for element concentrations among shrub organ (leaves, stem, and roots) as dependent variables, and soil water content at depth of 0-20 cm (SWC_{20}), 20-40 cm (SWC_{40}), and 40-100 cm (SWC_{100}) and their interactions as independent variables. Significant statistics ($P < 0.05$) are highlighted in bold.

Dependent variables		Independent variables						
Organ	Element concentration	SWC_{20}[1] *	SWC_{40}[2] *	SWC_{100}[3] *	1^2 *	1^3 *	2^3 *	1^2^3
Leaf	N	**F = 9.62**<br>***P* = 0.002**	**F = 21.3**<br>***P* <0.0001**	**F = 6.53**<br>***P* = 0.01**	**F = 4.67**<br>***P* = 0.03**	**F = 6.58**<br>***P* = 0.01**	(No significant)	(No significant)
	P	**F = 9.18**<br>***P* = 0.003**	**F = 18.6**<br>***P* <0.0001**	**F = 7.28**<br>***P* = 0.008**	**F = 3.92**<br>***P* = 0.04**	**F = 4.83**<br>***P* = 0.03**	(No significant)	(No significant)
	K	F = 0.37<br>*P* = 0.55	**F = 5.51**<br>***P* = 0.02**	F = 1.86<br>*P* = 0.17	**F = 10.5**<br>***P* = 0.001**	**F = 4.78**<br>***P* = 0.03**	(No significant)	(No significant)
	Na	F = 0.64<br>*P* = 0.42	**F = 4.26**<br>***P* = 0.04**	F = 0.007<br>*P* = 0.93	**F = 3.86**<br>***P* = 0.05**	(No significant)	(No significant)	(No significant)
	Ca	F = 1.58<br>*P* = 0.21	**F = 5.46**<br>***P* = 0.02**	F = 0.75<br>*P* = 0.39	(No significant)	(No significant)	(No significant)	(No significant)
	Mg	**F = 4.52**<br>***P* = 0.03**	**F = 9.60**<br>***P* = 0.002**	**F = 9.15**<br>***P* = 0.003**	(No significant)	**F = 10.1**<br>***P* = 0.002**	(No significant)	(No significant)
	Mn	F = 2.06<br>*P* = 0.15	**F = 6.60**<br>***P* = 0.01**	F = 1.34<br>*P* = 0.25	(No significant)	**F = 3.81**<br>***P* = 0.05**	(No significant)	(No significant)
	Zn	F = 0.61<br>*P* = 0.44	**F = 7.04**<br>***P* = 0.009**	**F = 6.23**<br>***P* = 0.01**	(No significant)	**F = 3.83**<br>***P* = 0.05**	(No significant)	(No significant)
	Cu	F = 0.58<br>*P* = 0.45	**F = 6.41**<br>***P* = 0.01**	**F = 4.79**<br>***P* = 0.03**	(No significant)	**F = 4.25**<br>***P* = 0.04**	(No significant)	(No significant)
	Fe	F = 1.69<br>*P* = 0.20	**F = 4.76**<br>***P* = 0.03**	F = 1.14<br>*P* = 0.29	**F = 3.91**<br>***P* = 0.04**	(No significant)	(No significant)	(No significant)
Stem	N	**F = 15.6**<br>***P* = 0.0001**	**F = 21.9**<br>***P* <0.0001**	**F = 4.27**<br>***P* = 0.04**	(No significant)	**F = 5.91**<br>***P* = 0.02**	(No significant)	(No significant)
	P	**F = 17.2**<br>***P* <0.0001**	**F = 22.5**<br>***P* <0.0001**	**F = 4.65**<br>***P* = 0.03**	(No significant)	**F = 3.89**<br>***P* = 0.05**	(No significant)	(No significant)

	K	F = 0.04<br>*P* = 0.84	**F = 1.54**<br>***P* = 0.22**	F = 0.01<br>*P* = 0.91	(No significant)	(No significant)	(No significant)	(No significant)
	Na	F = 1.46<br>*P* = 0.23	F = 3.48<br>*P* = 0.06	F = 0.15<br>*P* = 0.70	**F = 3.86**<br>***P* = 0.05**	**F = 12.0**<br>***P* = 0.0007**	(No significant)	(No significant)
	Ca	F = 0.67<br>*P* = 0.42	**F = 4.95**<br>***P* = 0.03**	F = 1.05<br>*P* = 0.31	(No significant)	(No significant)	(No significant)	(No significant)
	Mg	F = 0.28<br>*P* = 0.59	F = 1.68<br>*P* = 0.20	F = 2.34<br>*P* = 0.13	(No significant)	(No significant)	(No significant)	(No significant)
	Mn	F = 0.42<br>*P* = 0.52	**F = 5.19**<br>***P* = 0.02**	F = 1.17<br>*P* = 0.28	(No significant)	(No significant)	(No significant)	(No significant)
	Zn	F = 0.72<br>*P* = 0.40	**F = 7.03**<br>***P* = 0.009**	**F = 3.86**<br>***P* = 0.05**	(No significant)	(No significant)	(No significant)	(No significant)
	Cu	F = 0.73<br>*P* = 0.39	**F = 12.4**<br>***P* = 0.0005**	**F = 9.49**<br>***P* = 0.002**	**F = 4.05**<br>***P* = 0.04**	**F = 6.64**<br>***P* = 0.01**	(No significant)	(No significant)
	Fe	**F = 4.21**<br>***P* = 0.04**	**F = 4.87**<br>***P* = 0.03**	F = 0.58<br>*P* = 0.45	(No significant)	(No significant)	**F = 7.87**<br>***P* = 0.006**	**F = 6.19**<br>***P* = 0.01**
Root	N	**F = 11.7**<br>***P* = 0.0008**	**F = 29.6**<br>***P* <0.0001**	**F = 8.31**<br>***P* = 0.004**	(No significant)	**F = 8.90**<br>***P* = 0.003**	(No significant)	(No significant)
	P	**F = 8.92**<br>***P* = 0.003**	**F = 23.4**<br>***P* <0.0001**	**F = 8.51**<br>***P* = 0.004**	(No significant)	**F = 6.88**<br>***P* = 0.009**	(No significant)	(No significant)
	K	F = 0.23<br>*P* = 0.63	F = 1.04<br>*P* = 0.31	F = 0.11<br>*P* = 0.74	(No significant)	**F = 7.97**<br>***P* = 0.005**	(No significant)	(No significant)
	Na	F = 0.66<br>*P* = 0.42	**F = 7.20**<br>***P* = 0.008**	F = 0.81<br>*P* = 0.37	**F = 5.50**<br>***P* = 0.02**	**F = 24.4**<br>***P* <0.0001**	(No significant)	(No significant)
	Ca	F = 0.41<br>*P* = 0.52	**F = 3.87**<br>***P* = 0.05**	F = 0.94<br>*P* = 0.33	(No significant)	(No significant)	(No significant)	(No significant)
	Mg	F = 0.36<br>*P* = 0.55	F = 1.40<br>*P* = 0.24	F = 2.18<br>*P* = 0.14	(No significant)	(No significant)	(No significant)	(No significant)
	Mn	F = 0.20<br>*P* = 0.66	F = 2.67<br>*P* = 0.10	F = 1.36<br>*P* = 0.25	(No significant)	(No significant)	(No significant)	(No significant)
	Zn	F = 0.48<br>*P* = 0.49	F = 3.60<br>*P* = 0.06	F = 2.44<br>*P* = 0.12	(No significant)	(No significant)	(No significant)	(No significant)

	Cu	F = 1.44<br>$P = 0.23$	**F = 11.6**<br>**$P = 0.0008$**	**F = 8.97**<br>**$P = 0.003$**	**F = 3.80**<br>**$P = 0.05$**	**F = 7.46**<br>**$P = 0.007$**	(No significant)	(No significant)
	Fe	F = 0.22<br>$P = 0.64$	F = 1.84<br>$P = 0.18$	F = 2.48<br>$P = 0.12$	(No significant)	(No significant)	(No significant)	(No significant)

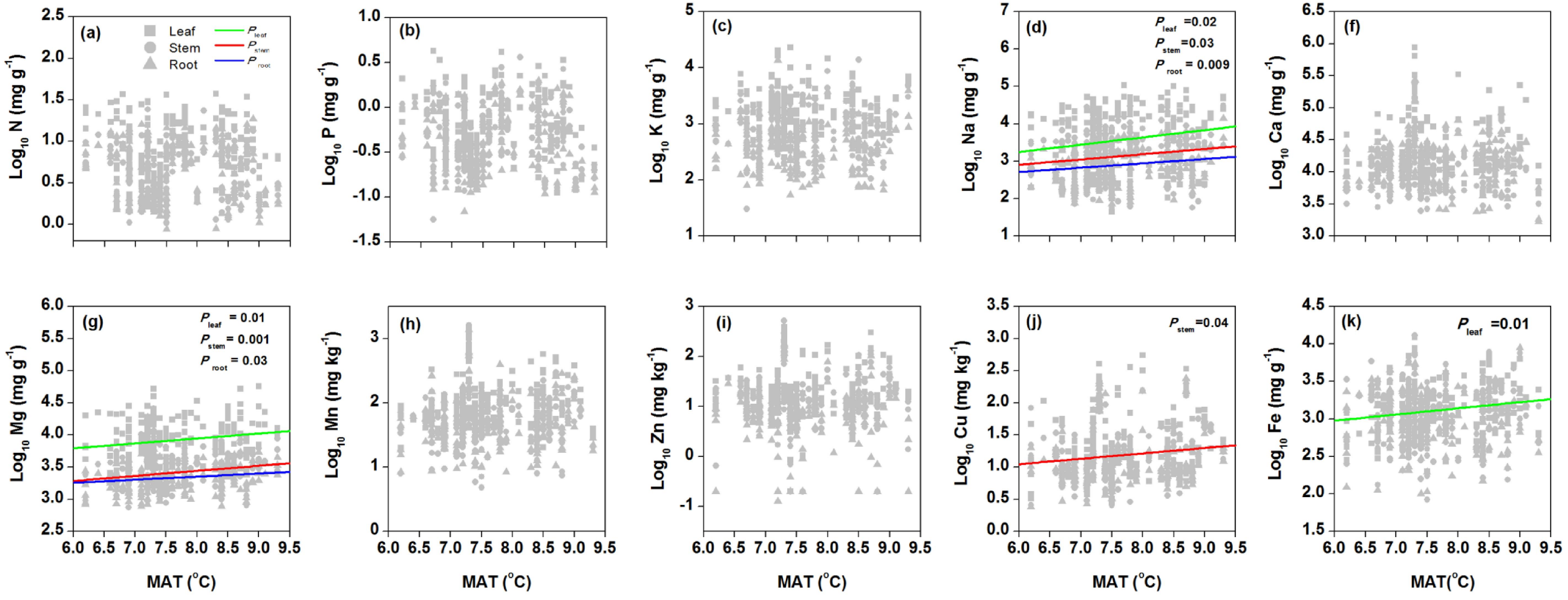

Figure S1 Relationships of mean annual temperature (MAT) with element concentrations (N, P, K, Na, Ca, Mg, Mn, Zn, Cu and Fe) among leaves, stems and roots. Colored lines represent significant relationships ($P < 0.5$) for shrub organ (green, leaves; red, stems; blue, roots).
